# Circulating leptin and adiponectin are associated with insulin resistance in healthy postmenopausal women with hot flashes

**DOI:** 10.1371/journal.pone.0176430

**Published:** 2017-04-27

**Authors:** Wan-Yu Huang, Chia-Chu Chang, Dar-Ren Chen, Chew-Teng Kor, Ting-Yu Chen, Hung-Ming Wu

**Affiliations:** 1 Institute of Basic Medical Sciences, College of Medicine, National Cheng Kung University, Tainan, Taiwan; 2 Department of Nephrology, Changhua Christian Hospital, Changhua, Taiwan; 3 Comprehensive Breast Cancer Center, Changhua Christian Hospital, Changhua, Taiwan; 4 Internal Medicine Research Center, Changhua Christian Hospital, Changhua, Taiwan; 5 Inflammation Research & Drug Development Center, Changhua Christian Hospital, Changhua, Taiwan; 6 Department of Neurology, Changhua Christian Hospital, Changhua, Taiwan; 7 Graduate Institute of Acupuncture Science, China Medical University, Taichung, Taiwan; University of California San Diego, UNITED STATES

## Abstract

**Introduction:**

Hot flashes have been postulated to be linked to the development of metabolic disorders. This study aimed to evaluate the relationship between hot flashes, adipocyte-derived hormones, and insulin resistance in healthy, non-obese postmenopausal women.

**Participants and design:**

In this cross-sectional study, a total of 151 women aged 45–60 years were stratified into one of three groups according to hot-flash status over the past three months: never experienced hot flashes (Group N), mild-to-moderate hot flashes (Group M), and severe hot flashes (Group S). Variables measured in this study included clinical parameters, hot flash experience, fasting levels of circulating glucose, lipid profiles, plasma insulin, and adipocyte-derived hormones. Multiple linear regression analysis was used to evaluate the associations of hot flashes with adipocyte-derived hormones, and with insulin resistance.

**Settings:**

The study was performed in a hospital medical center.

**Results:**

The mean (standard deviation) of body-mass index was 22.8(2.7) for Group N, 22.6(2.6) for Group M, and 23.5(2.4) for Group S, respectively. Women in Group S displayed statistically significantly higher levels of leptin, fasting glucose, and insulin, and lower levels of adiponectin than those in Groups M and N. Multivariate linear regression analysis revealed that hot-flash severity was significantly associated with higher leptin levels, lower adiponectin levels, and higher leptin-to-adiponectin ratio. Univariate linear regression analysis revealed that hot-flash severity was strongly associated with a higher HOMA-IR index (% difference, 58.03%; 95% confidence interval, 31.00–90.64; *p* < 0.001). The association between hot flashes and HOMA-IR index was attenuated after adjusting for leptin or adiponectin and was no longer significant after simultaneously adjusting for leptin and adiponectin.

**Conclusion:**

The present study provides evidence that hot flashes are associated with insulin resistance in postmenopausal women. It further suggests that hot flash association with insulin resistance is dependent on the combination of leptin and adiponectin variables.

## Introduction

Hot flashes are the most common bothersome symptoms during menopause. Hot flashes may happen during the day or at night (also known as night sweats). Up to 80% of women experience hot flashes during the menopause transition, and more than two-thirds during the post-menopause period, with most women rating hot-flash severity as moderate or severe [1]. At least half of those women experience frequent hot flashes lasting for more than seven years during the menopause [1]. Hot flashes and night sweats have a strong impact on sleep, mood, and cognitive function [2,3].

Despite hot flashes being considered a negative factor for quality of life during the climacteric period, hot flashes have generally not been assumed to have an impact on physical health. However, an increasing number of large clinical and epidemiological studies have countered that assumption [4,5]. Findings from a large trial involving hormone therapy initially revealed links between hot flashes and cardiovascular disease risk [6]. In that study, moderate to severe hot flashes reported at study entry among postmenopausal women receiving hormone replacement therapy were shown to be associated with increased risk of cardiovascular disease. The Study of Women’s Health Across the Nation and other studies further explored the potential links between hot flashes and cardiovascular disorders by studying subclinical risk factors for meta-cardiovascular disorders such as impaired lipid profiles and endothelial dysfunction in menopausal women [7–9]. The results of those studies provided a body of evidence suggesting that hot flashes may be closely linked to the development of cardiovascular disease.

Cardiovascular disease is the leading cause of death in the United States and in other developed countries [10]. Coincidently, cardiovascular risk increases in women after menopause, possibly due to estrogen deprivation or to the substantial metabolic changes that occur as women transition from a premenopausal to a postmenopausal state [11,12]. Insulin resistance is generally considered to be a risk factor for cardiovascular disease. Recently, both hot flashes and night sweats have been demonstrated to be strongly associated with insulin resistance as assessed by homeostatic model assessment in women undergoing the menopausal transition [4]. Adipose tissue is now recognized as an active metabolic and endocrine organ that regulates various metabolic functions. The adipocyte-derived hormones leptin, adiponectin, and resistin are known to play an important role in the development of insulin resistance, the main pathologic mechanism of many metabolic and vascular diseases [13]. These hormones have been postulated to play a potential role in hot flashes. However, few studies have evaluated their relationships. Sowers et al. found that the levels of adipocyte-derived hormones varied by menopausal stages and were possibly influenced by sex hormones (e.g. estrogen) [14]. Alexander et al. found that leptin was associated with duration and occurrence of hot flashes in relatively young middle-age women [15]. Thurston et al. found that lower adiponectin levels and higher leptin levels were marginally associated with higher odds of hot flashes in pre-/early perimenopause, but not in late peri-/postmenopause [16]. However, the evidence for an association between hot flashes with adipocyte-derived hormones provided in those studies is limited because the researchers did not fully evaluate the three hormones and because they did not control for potential confounding factors such as hot flash profiles (e.g. frequency and duration), sex hormones (e.g. estradiol and FSH), and systemic diseases (e.g. diabetes and hypertension).

Hot flashes are highly prevalent in women who are in the postmenopausal stage. In this population, however, the relationships between hot flashes, adipocyte-derived hormones, and insulin resistance have not been thoroughly investigated. We hypothesized that hot flash severity is associated with insulin resistance in postmenopausal women and that hot flash-associated insulin resistance is related to impaired adipocyte-derived hormones. To test our hypothesis, we compared lipid and metabolic profiles as well as adipocyte-derived hormone levels between groups of healthy postmenopausal women with and without hot flashes.

## Subjects and methods

### Participants and study design

In this cross-sectional study, women (aged 45 to 60 years) who visited the Changhua Christian Hospital for health management reasons during the period October 2012 to September 2015 were eligible for inclusion if they had a body-mass index (BMI) of more than 18 kg/m^2^ to less than 30 kg/m^2^, had at least 12 consecutive months of amenorrhea not due to surgery or other obvious causes, had never experienced hot flashes during menopause stages, or had experienced hot flashes within the three months prior to study entry. Women were excluded if they were in the early or late perimenopausal stage, were hormone therapy users, had undergone hysterectomy or bilateral oophorectomy, had a history of diabetes, hypertension (defined as blood pressure >140/90 mmHg or on antihypertensive medication), hyperlipidemia (defined as total cholesterol > 240 mg/dL or triglyceride > 200 mg/dL, or on statin medication) or thyroid disease, a history of smoking or had a BMI ≥ 30 kg/m^2^. The patient records and information were anonymized and de-identified prior to analysis. Written informed consent was obtained from all participants. This study was approved by the Changhua Christian Hospital Institutional Review Board (ID: CCH IRB No. 110305).

### Anthropometric measures

Blood specimens were collected from participants in the morning after an overnight fast at study entry. The plasma was aliquoted and stored at −80°C without thawing until assayed. Height and weight were measured in light clothing without shoes. BMI was calculated as weight (kg)/height (m)^2^ and considered primarily as a continuous variable.

### Hot flashes

In the present study, severity of hot flashes was clinically defined as: (1) mild when the flash was a sensation of heat without sweating; (2) moderate when the flash was a sensation of heat with sweating but did not interfere with daily activities; and (3) severe when the flash was a sensation of heat with sweating that caused cessation of activity during the day or interrupted sleep at night (also known as night sweats). If hot flashes occurred and persisted within the three-month period prior to study entry, women were asked to fill out a self-reported hot flash diary in which they were to report the total number of hot flashes they characterized as mild, moderate or severe over a two-week period [17]. The data on hot flashes were reported as mean frequency of hot flash severity per day during the two-week period. Severe hot flashes and night sweats have a negative impact on quality of life and can result in debilitating complications such as chronic insomnia [3]. In contrast, mild and moderate hot flashes are more or less tolerable and can be relieved by lifestyle changes. Accordingly, participants with mild to moderate hot flashes only were grouped together as one group and participants with severe hot flashes and/or night sweats were grouped together as one group. Women who fulfilled the inclusion criteria were divided into one of three groups based on the severity of hot flashes. Group N comprised postmenopausal women who had never experienced hot flashes or night sweats; Group M comprised women who had experienced mild-to-moderate hot flashes only at least four days per week; and Group S comprised women who had experienced severe hot flashes and/or night sweats at least once per day during the previous two-week period.

### Measurement of insulin and adipocyte-derived hormones

The levels of adiponectin and resistin were measured in frozen plasma specimens using human adipokine multiplex assays (Milliplex MAP kits, EMD Millipore, Billerica, MA, USA),. The levels of plasma insulin and leptin were measured using human metabolic hormone multiplex assays (Milliplex MAP kits, EMD Millipore, Billerica, MA, USA). All analyses were performed by T.-Y.C. according to the manufacturer’s protocol. The results of these four factors were read using a Luminex 200 system with a dynamic range of ≥ 3.5 logs (Luminex, Austin, TX, USA). Values for adiponectin were reported as μg/ml and those for resistin and leptin were reported as ng/ml. Insulin levels were expressed as pg/ml. The lower limit values of detection were as follows (pg/ml): insulin (22.1), leptin (38.5), adiponectin (27), and resistin (6.4), respectively. Data on adipocytokines and metabolic hormones were collected and analyzed using an instrument equipped with MILLIPLEX Analyst software (EMD Millipore). The samples of insulin and leptin were run undiluted, and those of adiponectin and resistin were run diluted 1: 80 using the assay buffer. If the sample concentration was outside the linear range of detection, the samples were retested after optimal dilution. All samples were analyzed in duplicates. The intraassay laboratory coefficients of variation (CVs) were as follows (%): insulin (6.4), leptin (5.3), adiponectin (5.6), and resistin (4.2), respectively. All values of the interassay CVs of these four factors were less than 10%. For quality control purposes, two replicates of control samples were tested on each microplate in any given run.

### Measurement of sexual hormones and other measures

Serum total cholesterol, triglyceride, low-density lipoprotein cholesterol (LDL), high-density lipoprotein cholesterol (HDL), fasting glucose, estradiol and follicle-stimulating hormone (FSH) were measured using standard procedures at the Department of Laboratory Medicine, Changhua Christian Hospital. Brief, the levels of estradiol and FSH were measured in frozen plasma specimens using the Access Estradiol assay and the Access hFSH assay on the Beckman Access Immunoassay system with a dynamic range of ≥ 3.5 logs (Beckman Coulter, Fullerton, CA.). Values for estradiol were reported as pg/ml and those for FSH were reported as mIU/mL. The lower limit value of detection was 20 pg/ml for estradiol and was 0.2 mIU/mL for FSH, respectively. The samples of estradiol and FSH were run undiluted. None of the others were outside the linear-range detection of standard curve. All samples were analyzed in duplicates. For quality control purposes, two duplicates of commercial control samples were tested on each microplate in any given run. The interassay and intraassay laboratory coefficients of variation (CVs) were < 8% and 8.6% for estradiol, respectively, and were < 8% and 5.6% for FSH, respectively. Insulin resistance was assessed based on the homeostatic model assessment of insulin resistance (HOMA-IR) using the following formula: [fasting glucose (mmol/L) × insulin (μU/L)/22.5].

### Statistical analysis

Results are presented as median (IQR: interquartile range) or mean ± standard deviation. Differences between Group N, Group M, and Group S were tested by one-way ANOVA or the Kruskal–Wallis test (Table 1). Tukey’s post hoc tests were then performed to find significant differences between groups (Table 1). Correlations were determined using Pearson’s correlation analysis. The associations between hot flashes and adipocyte-derived hormones were determined by multivariate linear regression after adjusting for BMI, FSH, age and menopause duration (Table 2). The association between hot flashes, adipocyte-derived hormones and HOMA-IR index was determined by univariate and multivariate linear regression analysis (Table 3). The percentage difference in each variable was calculated using the formula (100*(exp(β)-1)) and 95% CI for interpreting coefficients in the linear regression model. Effect size (Cohen’s f value) was calculated to identify the differences between the three groups (total sample size n = 151), and then power analysis was performed at type one error 0.05 using G*Power software 3.1. Statistical analyses were performed with SPSS software version 19.0.0 (IBM Corporation, Somers, NY, USA). A two-tailed *p* value < 0.05 was considered to be statistically significant.

**Table 1:** Characteristics of the participants by hot flash profiles.

Parameters	Hot flash status			*P* Value
	None	Mild to moderate	Severe	
n	52	47	52	
Age, years	55 (51.5, 58.0)	53 (51, 56)	53 (51.0,55.5)	.074†
MP_duration, years	4.0 (2.0, 5.0)	2.0 (1.7, 4.0)	2.5 (2.0, 5.0)	.054†
SBP, mmHg	110 (98, 115)	112 (96, 116)	114 (102, 120)	.065†
DBP, mmHg	71 (64, 75)	73 (64, 75)	72 (66, 75)	.592†
BMI, kg/m^2^	22.8 ± 2.7	22.6 ± 2.6	23.5 ± 2.4	.175‡
FSH, mIU/mL	69 ± 24	67 ± 25	65 ± 21	.316‡
Estradiol, pg/mL	< 20	< 20	< 20	—
Fasting glucose, mg/dL	92 ± 9	95 ± 7	99 ± 7*	< .0001‡
Hemoglobin A1c, %	5.5 (5.2, 5.6)	5.4 (5.2, 5.7)	5.6 (5.25, 5.8)	.189†
Total cholesterol, mg/dL	209 (191, 228)	202 (173, 223)	201.5 (182, 236)	.291†
Triglyceride, mg/dL	110 (84, 144)	97 (72, 144)	126 (81, 192)	.090†
HDL cholesterol, mg/dL	56 (46, 68)	52 (46, 58)	52 (44, 62)	.345†
LDL cholesterol, mg/dL	126 (104, 145)	123 (101, 139)	125 (103, 142)	.662†
Insulin, pg/ml	339 (261, 469)	394 (256, 509)	515 (388, 732)*	< .0001†
Leptin, ng/mL	9.2 (4.9, 11.5)	10.2 (7.4, 15.6)	16.2 (10.3, 20.6)*	< .0001†
Adiponectin, ug/mL	14.9 (11.3, 21.9)	14.7 (8.3, 29.0)	8.1 (6.3, 11.9)*	< .0001†
Leptin to Adiponetin ratio	0.63 (0.25, 1.09)	0.82 (0.36, 1.63)	1.73 (1.08, 2.84)*	< .0001†
Resistin, ng/mL	16.2 (12.6, 22.8)	15.8 (11.6, 21.1)	13.5 (11.3, 18.4)	.095†
HOMA-IR	1.20 (0.90, 1.84)	1.56 (1.00, 2.11)	2.13 (1.64, 2.97)*	< .0001†

Data are presented as mean ± SD or median (Q1, Q3). Statistical analysis was conducted by ANOVA test (marked with ‡) or Kruskal-Wallis test (marked with †) to compare the mean/median differences between three groups of postmenopausal women with or without hot flashes. Tukey’s post hoc tests were then performed to find significant differences between groups.

*, significant difference between Group S and Group M (*p* < 0.05) and Group S and Group N (*p* < 0.001).

Abbreviations: Q, quarter; Q1, 25^th^ percentile; Q3, 75^th^ percentile; MP_duration, menopause period since final menstrual period; SBP, systolic blood pressure; DBP, Diastolic blood pressure; FSH, follicle stimulating hormone; BMI, body mass index; HDL, high density lipoprotein; LDL, low density lipoprotein; HOMA-IR, homeostatic model assessment of insulin resistance.

**Table 2:** Association of hot flash status with adipocyte-derived hormones and HOMA-IR.

Variables	Leptin	Adiponectin	Resistin	Leptin/Adiponectin Ratio	HOMA-IR index
Hot flashes					
None					
Mild to moderate	25.79	-11.36	-6.67	51.03	12.04
	(4.54,51.36)^a^	(-30.13,12.44)	(-20.56,9.65)	(1.89,123.87)^a^	(-7.15,35.19)
Severe	53.18	-56.46	-16.86	140.29	53.89
	(29.78,80.79)^c^	(-71.33,-33.86)^c^	(-29.76,-1.59)	(70,239.64)^c^	(28.16,84.79)^c^

Data are expressed as the percentage difference (95% CI). Adipocyte-derived hormones and leptin/adiponectin ratio were log-transformed.

Regression coefficients were back-transformed using formula (100*(exp(β)-1)) to calculate the percentage difference and the 95% CI in each adipocyte-derived hormone for hot-flash group relative to non-hot flash group.

Multivariate model was adjusted for hot flashes, follicle stimulating hormone, body mass index, age, and menopause duration

^a^ P < .05

^c^ P < .001.

**Table 3:** Association of HOMA-IR index with hot flash status and adipocyte-derived hormones.

Variable	HOMA-IR index			
	Model 1	Model 2	Model 3	Model 4
Hot flashes				
None				
Mild to moderate	10.83(-8.58,34.36)	3.79 (-13.27,24.2)	0.85 (-15.35,20.15)	1.15 (-15.12,20.55)
Severe	58.03(31.00,90.64)^c^	29.13 (6.99,55.85)^b^	18.22 (-2.32,43.09)	19.09 (-1.82,44.45)
Leptin		3.3 (1.85,4.77)^c^	3.07 (1.66,4.5)^c^	3.06 (1.65,4.49)^c^
Adiponectin^-1^			21.86 (7.45,38.2)^b^	22 (7.58,38.36)^b^
Resistin				0.23 (-0.72,1.19)

Data are expressed as the percentage difference (95% CI). HOMA-IR index was log-transformed.

Regression coefficients were back-transformed using the formula (100*(exp(β)-1)) to calculate the percentage difference and the 95% CI in HOMA-IR index for hot-flash group relative to non-hot flash group.

Model 1: univariate linear regression model for HOMA-IR index; Model 2: adjusted for hot flash status, follicle stimulating hormone, body mass index, age, and menopause duration, and Leptin; Model 3: adjusted for Model 2 plus adiponectin^-1^; Model 4: adjusted for Model 3 plus resistin.

Adiponectin^-1^, the inverse of adiponectin level

^b^ P < .01

^c^ P < .001.

## Results

A total of 151 participants fulfilled the entry criteria and were enrolled in this study. They were divided into one of three groups based on severity of hot flashes. Group N comprised 52 women who had never experienced hot flashes or night sweats, Group M comprised 47 women who had mild-to-moderate hot flashes (mean daily number of hot flashes episodes ± S.D., 2.45 ± 1.48/day), and Group S comprised 52 women who had severe hot flashes and/or night sweats (4.48 ± 1.89/day) plus mild-to-moderate hot flashes (2.39 ± 0.89/day). There were no significant differences between the groups in median age, number of postmenopausal years since the last menstrual period, blood pressure, BMI or lipid profiles including total cholesterol, triglyceride, HLD-C, and LDL-C (Table 1).

One-way analysis of variance revealed that there were statistically significant differences in plasma levels of fasting glucose, insulin, leptin, and adiponectin, HOMA-IR index, and leptin to adiponectin ratio (all *p* values < 0.0001) among these three groups (Table 1). Tukey’s *post hoc* tests further revealed that women in Group S displayed significantly higher levels of leptin, fasting glucose, and insulin, and lower levels of adiponectin than those in Groups M (all *p* values < 0.05) and N (all *p* values < 0.001) (Table 1). There were no significant differences in values of those parameters between Group M and Group N (Table 1). In addition, all three groups had similar hemoglobin A1c levels and resistin levels.

Multivariate linear regression model was used to examine the relationships between hot flashes status and each of the three adipocyte-derived hormones after adjusting for follicle stimulating hormone, body mass index, age, and menopause duration. High leptin level and high ratio of leptin to adiponectin were significantly associated with both mild-to-moderate hot flashes (both *p* values < 0.05) and severe hot flashes (both *p* values < 0.001) and low adiponectin level was related to severe hot flashes (*p* < 0.001). Resistin level, however, was only marginally related to hot flash status.

**Fig 1:**
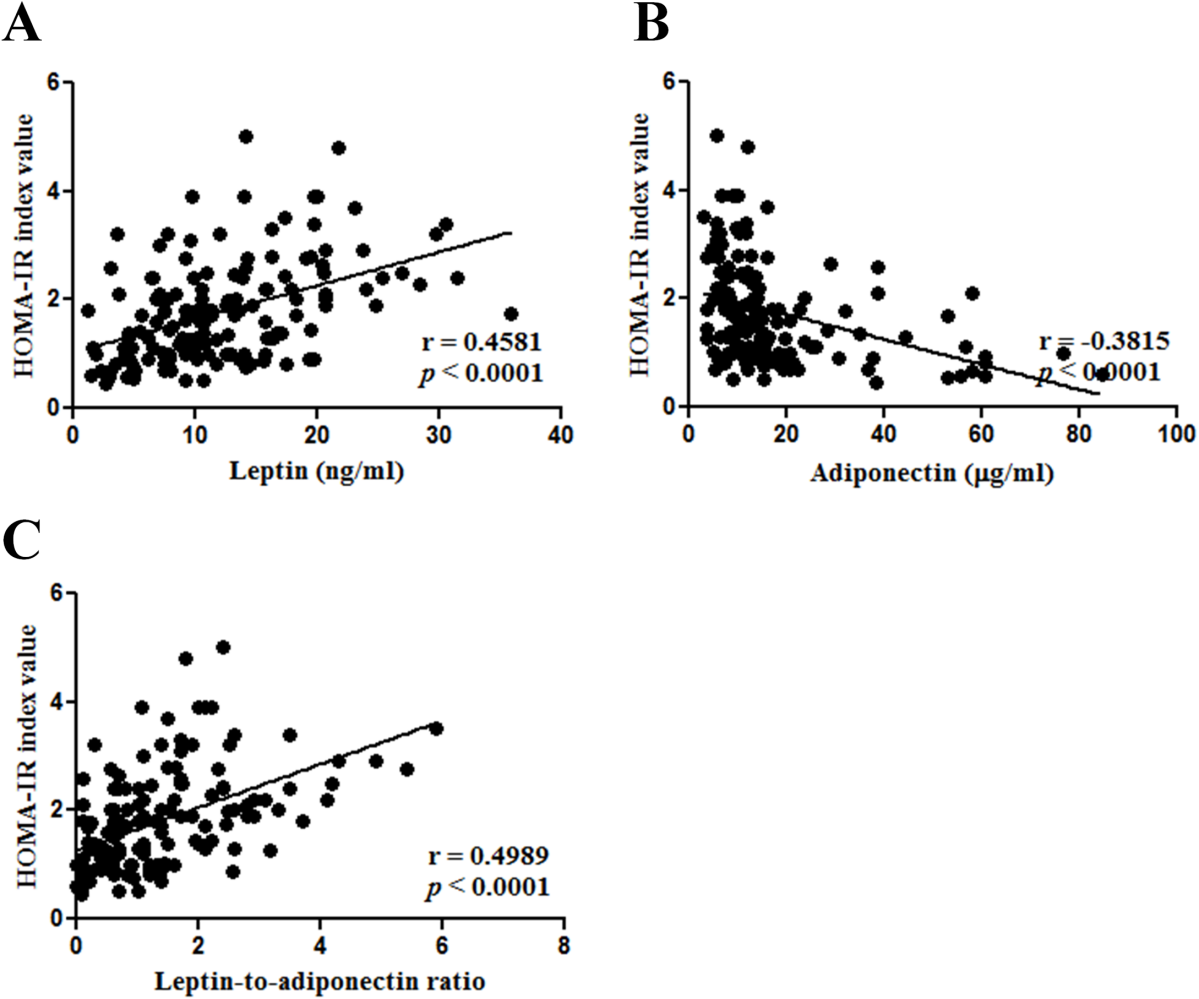
Associations of HOMA-IR index values with adipocyte-derived hormones. HOMA-IR index was correlated with leptin levels (A), adiponectin levels (B), and leptin-to-adiponectin ratio (C). Statistical analysis was conducted by Pearson’s correlation analysis.

Univariate analysis revealed that hot flashes severity had significant effects on HOMA-IR_log_ index (*p*_trend < 0.001). Compared to no hot flashes, severe hot flashes were strongly associated with a higher HOMA-IR_log_ index (% difference, 58.03%; 95% confidence interval (CI), 31.00–90.64; *p* < 0.001), whereas mild to moderate hot flashes were not (10.83%; 95%CI, -8.58–34.36; *p* > 0.05) (Table 3). Pearson’s correlation analysis showed a significant correlation between HOMA-IR index and overall plasma levels of leptin, adiponectin, and the ratio of leptin to adiponectin (all *P* values < 0.001) (Fig 1). HOMA-IR index were also positively correlated with BMI (*p* = 0.0448) and FSH levels (*p* = 0.0021). A number of studies have demonstrated that adipocyte-derived hormones play a role in insulin resistance [13]. Therefore, leptin and adiponectin were included in a multivariate linear regression model to identify the most important factors influencing the relationship between insulin resistance and hot flashes. The results revealed that the association between hot flashes and HOMA-IR was attenuated after adjusting for leptin (*P* < 0.001) or adiponectin (*P* < 0.01) (Table 3 and S1 Table). This association, however, was no longer significant after simultaneously adjusting for leptin and adiponectin. The effect size and power of those three variables were 0.412 and 99.6% for leptin and 0.363 and 96.9% for adiponectin.

## Discussion

In this study, we investigated the role of adipocyte-derived hormones in the relationship between vasomotor symptoms and insulin resistance in healthy postmenopausal women. We found that hot flash severity was significantly associated with elevated HOMA-IR index values and leptin-to-adiponectin ratio and that the association between hot flashes and HOMA-IR was significantly attenuated by leptin, adiponectin, or both, but not resistin. The results indicate that both leptin and adiponectin are linked to the association between hot flashes and insulin resistance in postmenopausal women.

Recently, Thurston et al. revealed a significant association between hot flashes and insulin resistance, as measured by HOMA-IR, in premenopausal and perimenopausal women [4]. In the present study, we found an association between hot flash-associated insulin resistance as measured by HOMA-IR and leptin-to-adiponectin ratio in postmenopausal women. Thurston et al, however, only examined the self-reported frequency of hot flashes in relation to HOMA-IR index [4]. In contrast, our participants provided at least a three-month history of hot flashes prior to study entry and those women who reported hot flashes recorded the frequency and severity of flashes in a daily diary. Since a number of characteristics of hot flashes including presence, frequency, duration, and severity may contribute important information about health outcomes in postmenopausal women, such as cardiovascular disease [18], our study is a more robust investigation of the relationship between hot flashes and insulin resistance.

A number of risk factors for the development of insulin resistance have been identified, mainly including BMI, hypertension, diabetes, diet, physical activity and age [19]. To control for these potential confounders, the present study only included participants without a history of hypertension, diabetes, hyperlipidemia, or other systemic disease. BMI and sex hormones are notably shared risk factors for hot flashes, adipocyte-derived hormones, and insulin resistance [4,15,16,19]. We therefore enrolled a relatively homogenous postmenopausal population to minimize variations of age, BMI, menopausal period and sex hormones (estradiol and FSH) (Table 1). Pearson’s correlation analysis showed that BMI (*P* < 0.05) and the sex hormone FSH (*P* < 0.05) were significantly related to HOMA-IR index, but their effects were not strong enough to attenuate the association of hot flashes with HOMA-IR index or with adipocyte-derived hormones and the leptin-to-adiponectin ratio (Table 2). Furthermore, the association of hot flash-insulin resistance was attenuated by leptin and adiponectin. These results therefore may add to the understanding of the mechanisms underlying the link between hot flashes and insulin resistance and no longer significant if accounting for both.

Thurston and the colleagues reported that adiponectin and leptin levels were related to hot flash status. High levels of leptin and low levels of adiponectin were observed in pre-menopausal and perimenopausal women with hot flashes compared to those without hot flashes [16].This inverse condition of plasma leptin and adiponectin was also observed in our postmenopausal women with hot flashes (Table 1). However, Thurston et al. found that the association of adipokines with hot flashes was marginally significant and varied by menopausal stage [16]. Accumulated evidence indicates that estrogen is an important shared risk factor relevant to hot flashes and adipocyte-derived hormones [15,16,20]. For instance, leptin concentrations vary with the level of sex hormones, particularly estrogen in ovulatory women [21,22]. In contrast, adiponectin is relatively stable and is not related to estrogen during the menstrual cycle [22]. Estrogen concentration in menopausal women fluctuates markedly during early and late perimenopause. Collectively this confounder may mask the subtle difference in adipokine levels in the perimenopausal stage between women with and those without hot flashes. In our postmenopausal participants, the levels of estradiol were undetectably low using our assay method. So we were unable to evaluate any confounding effect of estrogen on the association between hot flash status and the levels of leptin and adiponectin in our postmenopausal women (Table 2).

Adipocyte-derived hormones may affect insulin sensitivity by modulating insulin signaling pathways [23] and therefore may serve as potent indictors of the development of insulin resistance and risk for cardiovascular disease [24,25]. Adiponectin plays an important role in protecting against insulin resistance and inflammation, whereas leptin and resistin have the opposite effects [23,25]. Our participants with hot flashes had higher levels of leptin, but not resistin, and lower levels of adiponectin than women without hot flashes. Since leptin and adiponectin are secreted from adipose tissue, and resistin is produced mainly by macrophages [26], it is possible that adipose tissue dysfunction plays a role in hot flashes in menopausal women. The leptin-to-adiponectin ratio might, therefore, reflect the endocrine function of adipose tissue and provide a potentially useful measure of insulin resistance in subjects with obesity, metabolic syndrome, diabetes mellitus or non-diabetic mellitus [27–29]. In the present study, HOMA-IR index was strongly correlated with leptin, and adiponectin. Furthermore, the relationships between hot flashes and HOMA-IR were almost attenuated by leptin or leptin plus adiponectin. These results suggest that leptin and adiponectin are important mediators of hot flashes. When considered together, this adipokine-hot flash relationship in postmenopausal women is independent of insulin resistance (HOMA-IR).

There are several limitations to our study that need to be addressed. First, the cross-sectional design of this study did not allow us to determine whether a causal relationship exists between hot flashes, adipocyte-derived hormones, and insulin resistance. Second, the BMI values were similar among the three groups and more than three-fourths of the women had normal BMI values. Therefore, the results may not be applicable to other populations (e.g. obese groups). Third, although our study considered daytime hot flashes as well as nighttime hot flashes, our data could not identify the individual effects of these vasomotor symptoms on metabolism. This may underestimate the effects of night sweats on insulin resistance as night sweats frequently affect or interrupt sleep, leading to insomnia and cardiovascular risk [3,30].

In summary, the present study provides evidence for the association between hot flashes and insulin resistance in the postmenopausal population. Leptin and adiponectin, both of which are adipocyte-derived hormones, significantly contribute to the relationship between hot flashes and subclinical insulin resistance. Our results suggest that adipose tissue dysfunction is linked to hot flash-associated insulin resistance in postmenopausal women. Further longitudinal studies are required to clarify the causal relationships.

## Supporting information

S1 Table Association of HOMA-IR index with hot flash status and adipocyte-derived hormones without adjustment for hot flash status, follicle stimulating hormone, body mass index, age, and menopause duration. (DOCX)
