# Supplementary material for: Circulating leptin and adiponectin are associated with insulin resistance in healthy postmenopausal women with hot flashes
Source: PLoS One. 2017 Apr 27;12(4):e0176430. doi: 10.1371/journal.pone.0176430 (PMC5407749; doi:10.1371/journal.pone.0176430)
Supplement: S1 Table — (DOCX) [file pone.0176430.s001.docx]

**S1Table. Association of HOMA-IR index with hot flash status and adipocyte-derived hormones without adjustment for hot flash status, follicle stimulating hormone, body mass index, age, and menopause duration**

| **Variable** | **HOMA-IR index** | | | |
| --- | --- | --- | --- | --- |
|  | **Model 1** | **Model 2** | **Model 3** | **Model 4** |
| Hot flashes |  |  |  |  |
| None |  |  |  |  |
| Mild to moderate | 10.83(-8.58,34.36) | 3.2(-13.93,23.73) | 0.31(-15.91,19.67) | 0.62(-15.4,19.68) |
| Severe | 58.03(31.00,90.64)**^c^** | 29.59(6.95,57.02)^b^ | 18.13(-2.68,43.38) | 19.28(-1.59,44.56) |
| Leptin |  | 2.97(1.77,4.19)^c^ | 2.65(1.47,3.84)^c^ | 2.65(1.49,3.82)^c^ |
| Adiponetin^-1^ |  |  | 23.74(9.05,40.4)^b^ | 23.85(9.4,40.21)^b^ |
| Resistin |  |  |  | 0.33(-0.62,1.29) |

Data are expressed as the percentage difference (95% CI). HOMA-IR index was log-transformed.

Regression coefficients are back-transformed using formula (100*(exp(β)-1)) to calculate the percentage difference and the 95% CI in HOMA-IR index for hot flash group relative to non-hot flash group.

Model 1: univariate linear regression model for HOMA-IR index; Model 2: adjusted for hot flashes and Leptin; Model 3: adjusted for Model 2 plus adiponetin^-1^; Model 4: adjusted for Model 3 plus resistin.

^b^ *p* < .01; ^c^ *p* < .001.
